# Bronchogenic Cyst at the Terminal Ileum Presenting as an Enteric Duplication: A Case Report

**DOI:** 10.1055/a-2888-9612

**Published:** 2026-07-17

**Authors:** Thilo Stolze, Larissa Seidmann, Asim Zouari, Stephan Rohleder

**Affiliations:** 1 Department of Pediatric Surgery 39068 University Medical Center of the Johannes Gutenberg University MainzMainz, Rhineland-PalatinateGermany; 2 Department of Pediatric Surgery 14903 Charité University Hospital BerlinBerlinGermany; 3 Institute of Pathology 39068 University Medical Center of the Johannes Gutenberg University MainzMainzGermany

**Keywords:** bronchogenic cyst, subdiaphragmatic, abdominal, ileal, duplication

## Abstract

Bronchogenic cysts are congenital foregut malformations most often found in the mediastinum or lung parenchyma. Abdominal locations are exceedingly rare and most commonly found in the left retroperitoneum. They may mimic other intra-abdominal masses and pose a preoperative diagnostic challenge. A 17-year-old female presented with 4 days of diffuse, mild abdominal pain. Ultrasound and MRI identified a 5.5 × 3.8 × 3.1 cm unilocular, hypoechoic cyst adjacent to the terminal ileum at the ileocecal valve. Preoperative differential diagnoses included an ileal duplication cyst. Single-incision laparoscopy was converted to a small open ileocecal resection. Histopathology revealed a bronchogenic cyst, lined with ciliated respiratory epithelium with focal non-keratinized squamous epithelium. The patient recovered uneventfully and remained asymptomatic at 3-week follow-up. Although rare and often detected incidentally, bronchogenic cysts should be considered in the differential diagnosis of abdominal cystic lesions. Complete surgical excision is recommended to establish the diagnosis, relieve symptoms, and prevent potential complications. To our knowledge, this is the first reported case of a bronchogenic cyst located at the terminal ileum.

## Introduction

Bronchogenic cysts are rare congenital malformations of the tracheobronchial tree. Together with congenital pulmonary airway malformations, pulmonary sequestrations, hybrid lesions, and other kinds of foregut duplication cysts, such as neurenteric and enteric cysts, bronchogenic cysts are classified as bronchopulmonary foregut malformations. These lesions most commonly arise in the mediastinum adjacent to the trachea or main bronchi, less often within the lung parenchyma, and only rarely at ectopic sites such as cutaneous, subcutaneous, or intra-abdominal locations. 1

They arise between the third and seventh week of embryogenesis as abnormal budding from the primitive foregut, which differentiates into the tracheobronchial tree ventrally and the esophagus dorsally. An abdominal location of a bronchogenic cyst is extremely unusual, and it is presumed to result from the migration of the budding of the tracheobronchial tree into the abdominal cavity before being “pinched off” by fusion of the pleuroperitoneal membranes, which form the diaphragm. 2 3 Thus, abdominal bronchogenic cysts are a rare occurrence and pose both an embryological and a diagnostic challenge, being often misdiagnosed as other intra-abdominal masses. We report a rare case of a bronchogenic cyst located at the terminal ileum in an adolescent female patient.

## Case Description

A 17-year-old female patient was referred to our hospital with diffuse, mild abdominal pain for the past 4 days. There was no additional history of abdominal pain, nausea, vomiting, melena, or diarrhea. Furthermore, the patient reported no fever, night sweats, or unintentional weight loss. Upon physical examination, the patient showed mild tenderness on palpation in all quadrants of the abdomen. There were no signs of intestinal obstruction, peritonitis, abdominal swelling, skin stigmata, or palpable enlarged lymph nodes.

**Fig. 1:**
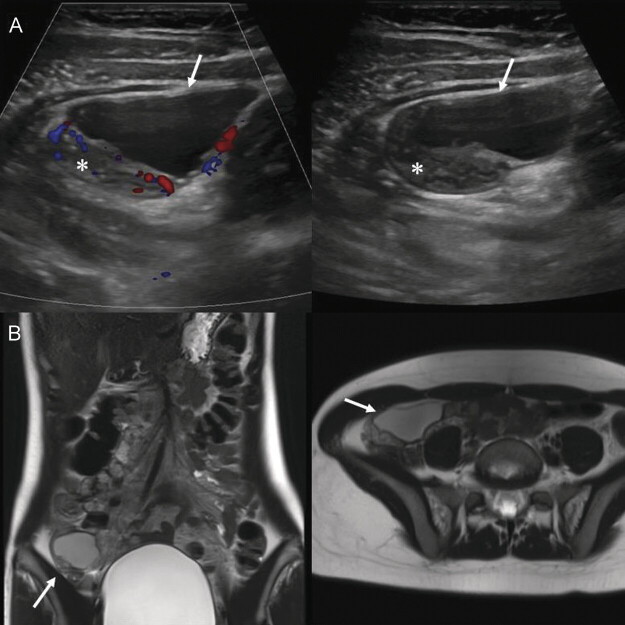
( **A** ) Transabdominal ultrasound showing a cystic mass (arrow) adjacent to the ileum (asterisk). ( **B** ) Abdominal MRI demonstrating a cystic mass (arrow) with smooth walls and homogeneous content.

Routine laboratory studies showed an elevated C-reactive protein (CRP, 47 mg/L), but a normal complete blood count. The tumor markers lactate dehydrogenase (LDH), α-fetoprotein (AFP), and human chorionic gonadotropin (hCG) were within normal range. Carbohydrate antigen 19-9 (CA19-9) was not assessed. Transabdominal ultrasound demonstrated a hypoechoic cystic mass measuring 5 × 4 × 2.5 cm in the right abdomen adjacent to the terminal ileum and the ileocecal valve ( Fig. 1A ). No solid components, relevant vascular perfusion, or connection to other organs, such as the right ovary, were identified. The subsequent MRI scan of the abdomen confirmed a cystic mass measuring 5.5 × 3.8 × 3.1 cm at the terminal ileum, immediately at the ileocecal valve, exhibiting a smooth wall and homogeneous, proteinaceous fluid content ( Fig. 1B ). No solid parts or enlarged lymph nodes were observed.

Based on these diagnostics, the preoperative differential diagnosis included a duplication cyst of the terminal ileum versus a neuroendocrine tumor. The case was reviewed by a multidisciplinary tumor board, which recommended primary surgical excision.

**Fig. 2:**
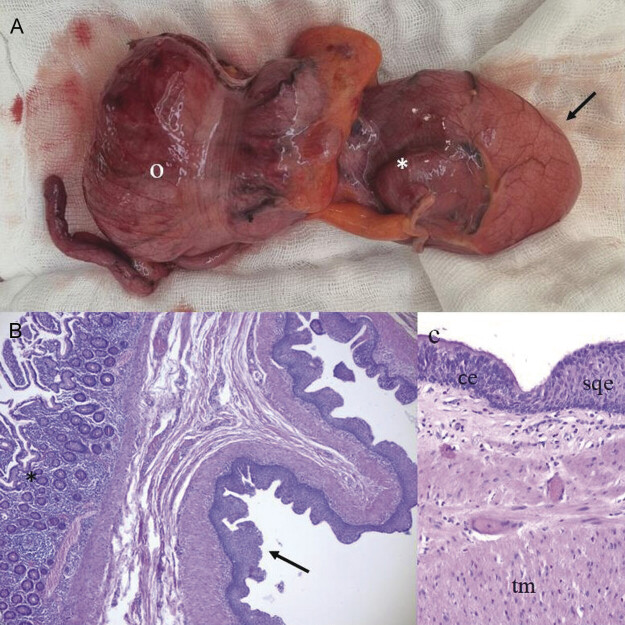
( **A** ) Resected specimen showing the bronchogenic cyst (arrow) adjacent to the terminal ileum (asterisk) in close proximity to the cecum (circle). ( **B** ) Histological assessment showing ileal mucosa (asterisk) opposite the lining of the bronchogenic cyst (arrow). ( **C** ) The bronchogenic cyst is lined with ciliated epithelium (ce) and squamous epithelium (sqe), adjacent to the tunica muscularis (tm) of the ileum.

Following the completion of preoperative diagnostic procedures, the cystic tumor was surgically approached by an umbilical single-incision laparoscopy. Given the risk of malignancy and intraoperative rupture, the procedure was converted to a small open approach to exteriorize the ileocecal segment. An ileocecal resection was performed using a linear stapler, maintaining a 3-cm macroscopic margin, followed by an end-to-end anastomosis. The unilocular cyst manifested as a grayish-red and soft elastic lesion ( Fig. 2A ). The patient was able to maintain a regular diet and was discharged on the fourth postoperative day without complications.

Histopathological examination revealed a cyst predominantly lined with ciliated epithelium and focal non-keratinized squamous epithelium consistent with a bronchogenic cyst, showing no signs of malignancy ( Fig. 2B, C ). Immunohistochemical staining was not performed.

At the 3-week follow-up, the patient was asymptomatic with normal gastrointestinal function and had resumed all daily activities.

## Discussion

Ectopic bronchogenic cysts have been described in various extrathoracic locations, including the skin, subcutaneous tissue, shoulder, and neck. Subdiaphragmatic manifestations of bronchogenic cysts are exceedingly uncommon and most frequently reported as retroperitoneal masses. 3 Among these, subdiaphragmatic lesions predominantly localize to the left retroperitoneum, often in the perigastric region, adjacent to the left adrenal gland, or within the superior pancreatic body, as documented in the largest published series (76.5% retroperitoneal; 81% left-sided). 3 4 The embryogenesis of this pathology likely involves aberrant caudal migration of a budding of the primitive foregut, prior to pleuroperitoneal membrane fusion. 5

Subdiaphragmatic bronchogenic cysts are typically small in size, asymptomatic, and are usually detected incidentally; however, patients may occasionally present with epigastric or left upper quadrant discomfort. 5 In this particular case, the bronchogenic cyst was discovered during evaluation for diffuse abdominal pain of unclear etiology and recent onset.

Preoperative diagnosis of an abdominal bronchogenic cyst remains challenging. Elevated serum CA19-9 levels have been reported in several cases of bronchogenic cysts, and were observed to return to normal following surgical removal. The epithelial lining of bronchogenic cysts can be immunohistochemically stained positively for CA19-9. This suggests that serum CA19-9 measurement may support the preoperative diagnosis of bronchogenic cysts. 6 7 8 9 However, the prevalence and underlying mechanisms of this increase remain unclear and require further investigation. Endoscopic ultrasound-guided fine needle aspiration (EUS-FNA) can reveal a bronchogenic cyst prior to resection by identifying ciliated columnar epithelial cells, but it is only reasonable in selected cases. 3 The definitive diagnosis of a bronchogenic cyst requires histopathological examination and is confirmed by the cyst wall being lined with respiratory epithelium (pseudostratified ciliated columnar or cuboidal epithelium). 3

The differential diagnosis for a subdiaphragmatic bronchogenic cyst is broad, including teratoma, gastrointestinal stromal tumor, adrenal neoplasm, mucinous cystadenoma, pancreatic pseudocyst, ovarian cyst, enteric duplication cyst, Meckel's diverticulum, abscess, echinococcosis, and lymphangioma.

Although most patients remain asymptomatic, bronchogenic cysts can become clinically significant by compressing adjacent anatomical structures or through complications such as infection or perforation. Of adult patients with thoracic bronchogenic cysts who were primarily managed conservatively, 45% to 68% developed symptoms. 10 11 Bronchogenic cysts have been observed harboring malignancy in 0.7% to 2.3% of cases, usually in the cyst wall. 10 11 A cystic mass may also conceal a neoplasia of a different etiology.

Given the potential complications, to alleviate symptoms and to establish the definitive diagnosis, surgical excision remains the treatment choice for bronchogenic cysts in pediatric patients. While the natural course of subdiaphragmatic bronchogenic cysts is uncertain, postoperative outcomes are generally excellent. 3 9 12 13

To our knowledge, four cases of bronchogenic cysts found at the ileum or ileal mesentery have been reported to date, none adjacent to the terminal ileum or colon. 5 13 14 15 This underscores the rarity of the present case and its contribution to the literature.

## Conclusion

Subdiaphragmatic bronchogenic cysts, though rare, should be considered in the differential diagnosis of abdominal cystic lesions and may present with elevated serum CA19-9 levels. These lesions are often small, asymptomatic, and incidentally detected. The treatment of choice remains complete surgical excision to confirm the diagnosis, relieve symptoms, and prevent complications, including organ compression, infection, and the rare risk of malignant transformation.

## Notes

Importance for a Pediatric Surgeon Bronchogenic cysts can occur at unusual intra-abdominal locations, such as the terminal ileum, and closely mimic other cystic lesions, including duplication cysts and neuroendocrine tumors. This poses a significant preoperative diagnostic challenge. It is imperative to raise awareness of this rare entity to broaden the differential diagnosis of abdominal cystic masses, particularly in cases where elevated CA19-9 levels are present. Complete surgical excision remains both diagnostic and curative, thereby relieving symptoms and preventing complications such as compression, infection, or rare malignant transformation.
